# Three-level model for opinion aggregation under hesitance

**DOI:** 10.1007/s00500-023-07853-2

**Published:** 2023-02-10

**Authors:** František Zapletal, Miroslav Hudec, Miloš Švaňa, Radek Němec

**Affiliations:** 1 Department of Systems Engineering, Faculty of Economics, VŠB- Technical University of Ostrava, Sokolská 33, 70200 Ostrava, Czech Republic; 2 Department of Applied Informatics, Faculty of Economic Informatics, University of Economics in Bratislava, Dolnozemska cesta 1, 852 35 Bratislava, Slovakia

**Keywords:** Hesitance, Three-level aggregation, Fuzzy logic, Opinion collection, Relative quantifier, Choquet integral, Decision-making support

## Abstract

Valuable information for decision-making can be obtained by collecting and analyzing opinions from diverse stakeholder or respondent groups, which usually have different backgrounds and are variously affected by the topics under survey. For this to succeed, it is necessary to manage the uncertainty of respondents’ opinions, different number of filled questionnaires among groups, different number of questions for each stakeholder group, and relevance of subsets of respondent groups. This work proposes handling the hesitance of respondents’ opinions for the rating scale questions. To evaluate the collected opinions, a three-level aggregation model is developed. In the first level, the overall opinion of each respondent is computed as a mean of fuzzy numbers covering uncertain answers and their respective hesitance. In the second level, stakeholder groups are considered as a whole. Aggregation by a relative quantifier is applied to calculate the validity of a proposition *the majority of respondents have a positive or negative opinion*. At the third level, the consensus among diverse subsets of stakeholder groups is calculated considering the relevance of each group independently as well as their so-called coalitions by Choquet integral. Finally, the proposed model is illustrated by a real-life case study.

## Introduction

Valuable information for decision-making can be obtained by collecting opinions on various key issues among diverse stakeholder groups (e.g., in a smart city or in blended learning). Such surveys help to identify the directions in which these issues can be advanced. For instance, various investment projects in a city may be worthwhile based on facts and figures, but these do not provide information about the impact on the personal or professional lives of affected stakeholders who live or work at the site. It is likely that there will be different opinions among stakeholder groups (e.g., residents, commuters, ecologic activists, etc.). However, such opinions can help to indicate how to develop in a positive way.

Collecting stakeholder perceptions and experiences by surveys is a common practice, because it is expected that user experience is observable and measurable (Albert and Tullis 2013; Snijkers et al 2013). This usually requires the collection of qualitative rather than quantitative data. Assuming that a survey can be conducted in the context of a city to get opinions about an urban topic (e.g., creation of a new neighborhood, expansion of public transportation), or in the context of education affected by the pandemic situation (e.g., creating courses and teaching in specific platforms) the following issues can appear and should be considered:

- Groups of stakeholders with different levels of expertize and skills as well as own preferences and goals (in a city: residents, commuters, ecologic activists, experts in transportation, and so on; in online education: teachers, students, technical staff, etc.)
- Different sizes and backgrounds of these groups. The former causes differences in the number of questionnaires filled in by each group and therefore troubles with the answers aggregation. The latter can result in hesitation when providing categorical answers. In addition, questionnaires should be tailored to each group to improve the cooperation in surveys. However, such questionnaires might contain an unequal number of questions and different granulation of answers.
- Evaluation should be performed at individual level, as well as at the level of coalitions among respondent groups. Then, several coalitions can be more relevant than the others, or when the key groups agreed, it should be also emphasized.
- Some participants do not fill in the questionnaire carefully (e.g., the respondent selects the neighboring value).

To solve these issues, this paper focuses on the development of a robust aggregation model for evaluation.

The research question in this work is the following: Could hesitance in answers, aggregation of answers within non-balanced groups, and aggregation among groups (considering the relevance of a subset of groups, so-called coalitions) be solved by fuzzy logic and aggregation functions? By this approach, we can bridge the gap in the evaluation of opinions among diverse groups of respondents and manage opinions by fuzzy logic and logic aggregation functions.

In the literature, studies are focused on opinion collection and evaluation from smaller expert groups, or surveys considering the whole population (or a representative sample). The latter also covers the evaluation of opinions from larger groups of evaluators or respondents, e.g., by a web application for a higher number of respondents, and questionnaires, where respondents are asked to evaluate multiple alternatives (Morente-Molinera et al 2018). The granularity of answers might be different across diverse groups and subsequently converted to the basic granulation or basic linguistic term set (Morente-Molinera et al 2019) by, e.g., the approach developed in (Herrera and Martínez 2001). The three-level aggregation model, introduced in Rakovská and Hudec (2019), should be advanced to create an enhanced survey evaluation model capable to address the hesitance of respondents and handle different levels of relevance among the coalitions of respondents groups subsets.

To soften the gap in these fields, and to propose a possible solution, this work is focused on the development of a robust three-level model for evaluation and aggregation of opinions from different stakeholder groups under hesitance.

The main part of this paper is devoted to the formalization of uncertainty in individual answers, aggregation answers within a stakeholder group and among groups considering different relevance of subcategories of groups. The model is illustrated by an example conducted by a survey in the city of Ostrava in the Czech Republic. The main purpose was to illustrate our idea by demonstrating its applicability in a real-world situation.

The categorization of groups depends on the area where a survey is conducted to effectively address the problem or challenge. This categorization of stakeholders can be reinforced, for instance, by an illustrative and very current example: the survey of opinions on COVID-19 vaccination. One can find many different opinions on the risks and benefits of vaccination. Since the information available in the media can be highly biased, it is helpful to consider not only the opinions of medical experts, but also the opinions of other stakeholder groups, such as physicians, biologists, economists, highly sensitive groups of citizens, young citizens, etc. This heterogeneous opinion picture allows the authorities to better understand the needs, concerns, and motivations of various stakeholders and thereby develop a better vaccination strategy. Similar logic can be applied in other fields, such as the development of a new neighborhood, the construction of a new shopping center, or the closure of a store or school, where a survey with different stakeholder groups would also be beneficial. In this paper, this benefit is demonstrated by a case study concerning the construction of a new tram track.

The rest of the paper is organized as follows. Section 2 provides a thorough literature review on existing approaches to opinion aggregation and evaluation. In Sect. 3, the proposed evaluation model is carefully described together with the necessary methodological preliminaries. Section 4 demonstrates the application of the proposed model on the case study of urban development. Section 5 critically discusses the obtained results and the implications of the proposed model for a decision-maker. Section 6 concludes the paper and provides an outlook for further research.

## Managing opinions: state of the art

Whenever one deals with a decision-making problem with more decision-makers, two main questions have to be addressed: (1) how to evaluate the opinions of individual respondents and (2) how to aggregate all opinions to make a final decision.

### Evaluating opinions from different stakeholders

As already discussed, surveys are a common tool for gathering information to support decision-making. To capture an opinion of a respondent that is qualitative in nature, survey designers traditionally employ some form of a linguistic scale, most commonly a variant of the Likert scale. The survey respondent usually selects one answer from an ordered set of linguistic terms expressing various levels of attitude ranging from absolutely negative to absolutely positive.

The popularity of the Likert scale is supported by its many advantages. Johns (2010) mentions simplicity and versatility as its main advantages. Nemoto and Beglar (2014) provides a more extensive and detailed list of advantages: “(a) data can be gathered relatively quickly from large number of respondents, (b) they can provide highly reliable person ability estimates, (c) the validity of the interpretations made from the data they provide can be established through a variety of means, and (d) the data they provide can be profitably compared, contrasted, and combined with qualitative data-gathering techniques, such as open-ended questions, participant observation, and interviews”.

However, as pointed out by Li (2013a), the Likert scale also suffers from several disadvantages. First, it is unclear whether the scale is ordinal or interval. Given the scale is viewed as an interval, the second problem arises: is the scale equidistant, i.e., are the distances between neighboring choices always equal? Third, the closed response format might mean that some respondents cannot accurately express their opinion. These issues lead to information loss and/or information distortion. The last disadvantage gives rise to the need of considering uncertainty in evaluation.

The degree of uncertainty, or more generally the level of knowledge in input data is an important factor in decision-making. Besides the aforementioned issues, the level of completeness of all required evaluations has to be taken into account, as some information might not be available to the decision-makers (Zhou et al 2018). Instead of using precise real numbers, one can consider alternative expressions that are able to incorporate various forms of uncertainty: random variables, intervals, fuzzy sets or their various extensions.

Various sources of uncertainty can be considered when modeling a group decision-making problem. One of them stems from the necessary aggregation of the individual evaluations, see Sect. 3.5.1, i.e., the uncertainty in the final judgement is caused by the variability of the individual evaluations. For instance, Lalla et al (2005) used the (precise) individual evaluations to establish the uncertain, fuzzy evaluation of the whole group (see the brief description of the fuzzy approach in Sect. 3.1). Based on the frequency of particular evaluations, random variables can also be used to get the grouped assessment (Lahdelma et al 1998). However, even the individual evaluations can be considered imprecise. The linguistic Likert scale captures a qualitative measure: opinion. As already mentioned, it is not always easy to assign one particular degree from the scale to express the opinion. Fourali (1997) presented an approach where the respondents choose intervals instead of just one grade from the used scale. The Likert scale is also often fuzzified and each precise degree is replaced by the corresponding imprecise (fuzzy) set, see Chen et al (2015); Sun (2010); Wicher et al (2019). However, more creative fuzzy extensions also exist. Li (2013a) asked respondents directly to assign membership degrees to possible answers. Gil and González-Rodríguez (2012) let respondents draw the whole fuzzy sets (their membership degrees). Vonglao (2017) represented each answer by a bell-shaped fuzzy set stretching over the whole base interval. Moreover, respondent opinions are combined with aspects of discrimination and validity, and an expert system is constructed to evaluate the answers. Similarly, Árva et al (2019) used two conjoined sigmoids to define the membership functions of various linguistic terms, highlighting the benefit of easy descriptive statistical analysis. For sure, all the mentioned approaches can be useful in some situations.

Since the model presented in this paper does not aim only at experts - it should be suitable for everyone, it is necessary to choose a reasonable compromise between the amount of information and user friendliness. The amount of time needed to express individual opinions should not be exhaustively large and all questions should be naturally easy-to-understand. The proposed model utilizes fuzzy sets as a tool to capture answer hesitance/uncertainty. Intervals can be viewed as a special case of a fuzzy set, and, in our opinion, the stochastic approach is not very suitable for capturing the uncertainty in individual opinions. The approach using the fuzzified Likert scale (Li 2013b) is easy and fast to use, but does not allow a respondent to express the uncertainty individually (it just admits that two respondents choosing two neighboring values on the scale can have, to some extent, the same opinion). If respondents had been able to express their own uncertainty by constructing a fuzzy rating membership function (as proposed by Li (2013a); Gil and González-Rodríguez (2012), it would have been an ideal scenario. However, such a task is not trivial even for somebody with good knowledge of (fuzzy) mathematics. Instructions on how the opinion is expressed would be too exhausting for both respondents and interviewers. Therefore, we decided for the “middle” way. The fuzzy sets will be constructed automatically, but a respondent would be able to express to what extent he or she hesitates with the answer. The level of this hesitance will determine the shape of the resulting fuzzy evaluation. Thus, in this case the uncertainty is not caused by the scale itself, but by the level of knowledge and indecisiveness of the respondent.

### Aggregating opinions from different respondents or stakeholder categories

Gathering opinions from respondents can be viewed as a special case of a group decision-making problem, thus it is reasonable to explore the possibilities of evaluation aggregation in general. When dealing with any decision-making problem with more decision-makers (DMs), one has to always choose a suitable way of opinion aggregation. Only if the group of DMs is small and homogeneous enough, and if the structure of the given problem is identical for all DMs, the consensus vote can be used instead (Ishizaka and Nemery 2013) (all DMs discuss their opinions together and they behave like only one DM). Otherwise, the aggregation of individual opinions is inevitable. One can choose between several well-established multi-criteria decision-making methods (MCDM), in whose algorithms, the aggregation in case of more decision-makers is usually available. In MCDM methods, simple aggregating operators are usually used: geometrical mean (e.g., in AHP method (Ishizaka and Nemery 2013)), weighted mean or arithmetic mean (e.g., in TOPSIS method (Shih et al 2007)). Specialized consensus models often use more complex/generalized aggregations: various versions of OWA operators (Ordered Weighted Averaging operator (Bueno et al 2019; Chiclana et al 2001)), Sugeno integral (Couceiro et al 2016)), or Choquet integral (Zhu and Li 2018)). The Sugeno integral is applied for ordinal data, and the Choquet integral to cardinal data.

Another important issue related to the opinion aggregation should be taken into account. DMs usually have (at least a bit) different views on the relevance of each DM (or group of DMs) and the possible coalitions among them. Different relevance can be solved by assigning weights (Ishizaka and Nemery 2013) and performing aggregation by some operator from the OWA operators family (Chiclana et al 2001). Coalitions between DMs can give rise to synergies in the relevance of opinions. These synergies can be captured by the Choquet and Sugeno integrals already mentioned above. It is worth noting that the OWA operators are a particular case of discrete Choquet integral (Murofushi and Sugeno 1993).

## Three-level aggregation model for evaluating opinions

First, this section provides the necessary mathematical background. Then, in the main part of this section, the three-level aggregation model is proposed.

### Preliminaries of fuzzy set theory

Since the proposed methodology works with the uncertainty expressed by vague (fuzzy) data, some basics of the fuzzy sets approach are provided in this section.

A fuzzy set ${\mathcal {F}}$ is a subset of universe ${\mathbb {X}}$ described by the membership function $\mu _{{\mathcal {F}}}(x)$, which assigns the membership degree $\alpha$, $\alpha \in [0,1]$, to each $x\in {\mathbb {X}}$ (Zadeh 1965). The set of *x* for which the assigned membership degree is equal to 1 is called the core of the fuzzy set ($\text {Core}({\mathcal {F}})$). The set of *x* is for which the assigned membership degree is greater than zero is called the support of the fuzzy set ($\text {Supp}({\mathcal {F}})$).

**Fig. 1:**
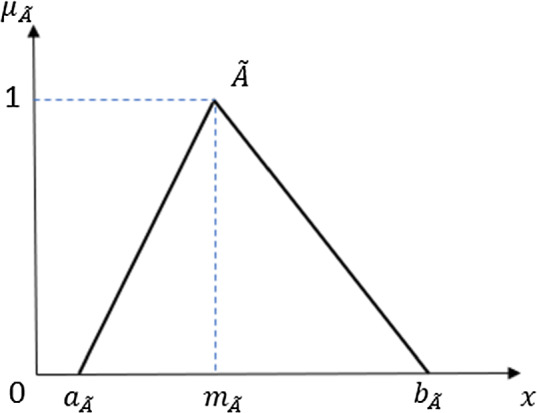
Triangular fuzzy number $\tilde{A}=(a_{\tilde{A}},m_{\tilde{A}},b_{\tilde{A}})$

**Fig. 2:**
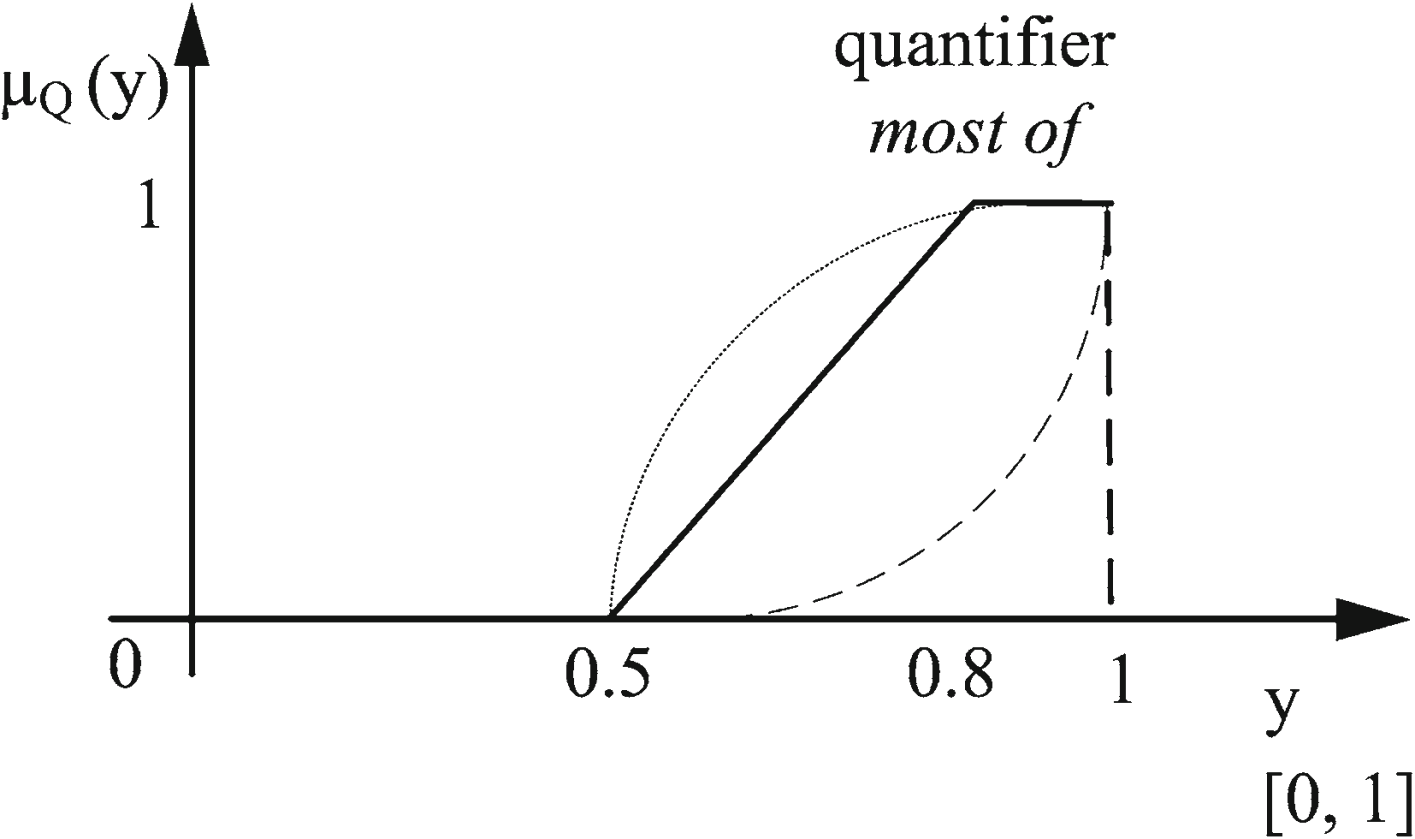
Quantifier *most of* expressed by linear and non-linear functions

In this paper, two types of fuzzy sets, whose membership functions are piecewise linear are used: triangular fuzzy numbers (TFN) (see Fig. 1) and linear gamma function (LGF) (Fig. 2). The former is used to capture the vagueness and hesitance of respondent opinions. It means the following: the answer is most likely $m_{\tilde{A}}$, but due to hesitance not lower than $a_{\tilde{A}}$ and not higher than $b_{\tilde{A}}$. The latter is applied for formalizing fuzzy relative quantifier *most of* and concepts like *positive opinion* (explained later on in Sect. 3.5.2).

Both these simple fuzzy sets have an advantage that the linearity makes the calculations easier. In particular, the operations of addition and multiplication between two fuzzy sets are necessary for aggregating respondents’ answers. If $\tilde{A}$ and $\tilde{B}$ are TFNs defined by the triplets $(a_{\tilde{A}},m_{\tilde{A}},b_{\tilde{A}})$ and $(a_{\tilde{B}},m_{\tilde{B}},b_{\tilde{B}})$, respectively (the notation is in line with Fig. 1), the fuzzy addition $\tilde{A}\oplus \tilde{B}$ and fuzzy multiplication $\tilde{A}\otimes \tilde{B}$ can be written as follows:

1 $$\begin{aligned} \tilde{A}\oplus \tilde{B}=(a_{\tilde{A}}+a_{\tilde{B}},m_{\tilde{A}}+m_{\tilde{B}},b_{\tilde{A}}+b_{\tilde{B}}), \end{aligned}$$

2 $$\begin{aligned} \tilde{A}\otimes \tilde{B}\approx (a_{\tilde{A}}\cdot a_{\tilde{B}},m_{\tilde{A}}\cdot m_{\tilde{B}},b_{\tilde{A}}\cdot b_{\tilde{B}}). \end{aligned}$$

The third operation required for the proposed model is the multiplication of a TFN by a real number, which can also be derived from (2):

3 $$\begin{aligned} p\cdot \tilde{A}=(p\cdot a_{\tilde{A}}, p\cdot m_{\tilde{A}}, p\cdot b_{\tilde{A}}), \end{aligned}$$

where $p\in {\mathbb {R}}^+$. For instance, arithmetic mean of TFNs is obtained by their sum (1) and consequently multiplication by $p=\frac{1}{n}$ where *n* is the number of TFNs.

A common way how to extend a crisp relation to a fuzzy one is the Zadeh’s extension principle (Ramík and Vlach 2012; Zadeh 1996). The possibility measure (Pos) builds on this extension principle and, moreover, provides a natural comprehensible interpretation. Namely, the possibility degree from [0, 1] expresses to what extent, it is possible that the given relation holds, for instance, that fuzzy set (in this case a TFN) $\tilde{A}$ belongs to the fuzzy set (or concept with the non-sharpened boundaries) $\tilde{B}$ (see Figs. 4 and 5 in Sect. 3.5.2):

4 $$\begin{aligned} \text {Pos}(\tilde{A},\tilde{B})= \sup _{x}\min \bigl ( \mu _{\tilde{A}}(x),\mu _{\tilde{B}}(x)\bigr ). \end{aligned}$$

The above introduced concepts are applied in the following sections.

### Aggregation by quantifiers

Aggregation by the relative quantifier *most of* reveals, whether the majority of entities, or respondents have considered values of attributes or opinions, respectively, for instance in the structure *the most of respondents in a group has positive opinion regarding the considered topic*.

A formal structure of the summarized sentence is *Q entities have P*, where *Q* is a linguistic quantifier and *P* is a crisp or fuzzy predicate. The validity of such sentence is calculated by Yager (1982):

5 $$\begin{aligned} v\Big ({Qx}\big ({P}({x})\big )\Big )\ =\ \mu _{Q}\bigg (\frac{1}{n}\ \sum _{i=1}^{n}\ \mu _{P}(x_i)\ \bigg ) \end{aligned}$$

where *n* is the number of entities (resp. respondents in a group), $y=\frac{1}{n}\sum _{i=1}^{n}{\mu _P(x_i)}$ is the proportion of respondents that satisfy predicate *P*, $\mu _Q$ and $\mu _P$ formalize the chosen relative quantifier (here *most of*) and predicate (here *positive opinion*), respectively, by membership functions.

The quantifier *most of* is an increasing function $\mu _Q(x):[0,1]\rightarrow [0,1]$ where $\mu _Q(0)=0$ and $\mu _Q(1)=1$ hold. According to Kacprzyk and Zadrozny (2005), it can be expressed as

6 $$\begin{aligned} \mu _Q\ (y)=\min \ \Bigg (1,\max \ \Big (0,\frac{y-0.5}{0.3}\Big )\Bigg ) \end{aligned}$$

where *y* is the proportion of respondents having positive opinion. A non-linear function covers further details like *significant majority is required* or *a weak majority suffices* (see Fig. 2 - dashed and densely dashed lines, respectively). A piecewise linear function is mainly adopted for the sake of simplicity.

Observe that any aggregation function should meet three axioms (Grabisch et al 2009): monotonicity, and two boundary conditions: $f(0,0,\ldots ,0)=0$ and $f(1,1,\ldots ,1)=1$. Clearly, when all respondents’ answers do not belong to the *positive opinion*, even partially, the result is 0. Opposite, when all answers express a clear positive opinion, the result is 1. When the number of positive answers increases, the result of aggregation either remains the same or increases. Thus, the monotonicity is met.

This aggregation way mitigates the influence of careless answers (i.e., respondents who provide neighboring values instead of desired ones are evaluated similarly). Next, it also deals with the aforementioned problem of unbalanced group sizes with different number of questions in each group (Rakovská and Hudec 2019).

### Aggregation by Choquet integral

When one wants to cover diverse situations in aggregation answers, the following issues can be considered:

- If we have equally important respondents or entity attributes, the arithmetic mean is used. This function is also considered as a logically neutral aggregation function (Dujmović 2018), i.e., a lower value for one attribute is compensated by a higher value of another one.
- When opinions or attributes are not equally important, we can aggregate them by the weighted arithmetic mean. It is still a logically neutral aggregation function, where weights meet the requirement: $\sum _{i=1}^{n}w_{i}=1$. This is not a suitable way when a higher number of attributes is evaluated. Observe that for $n=9$ where one attribute is twice more relevant than any of the others, we get, i.e., $w_{1}=0.2$ and $w_{2}=\ldots =w_{9}=0.1$. The value is multiplied by 0.2 and moreover the difference between 0.1 and 0.2 is relatively small.
- When several subsets of respondent groups (or attributes) are more relevant than the others, the usual averaging functions like arithmetic mean (and its weighted version) cannot solve this task.

The common solution for these cases is the Choquet discrete integral, which considers the intensities of all attributes and their relevance in various subsets.

Aggregation functions based on the discrete Choquet integral defined with respect to a measure belong to the class of averaging functions (Beliakov et al 2020, 2016). Choquet integration is based on not necessarily additive monotone measures (Choquet 1953) $v:2^{\mathcal {N}}\rightarrow [0,1]$. A discrete fuzzy measure (Wang and Klir 1992) is a set function on ${\mathcal {N}}=\{1,2,\ldots ,n\}$ which is monotonic ($v({\mathcal {A}})\le v({\mathcal {B}}$) whenever ${\mathcal {A}}\subseteq {\mathcal {B}}$) and satisfies boundary conditions $v(\emptyset )=0$ and $v({\mathcal {N}})=1$.

A subset ${\mathcal {A}}\subseteq {\mathcal {N}}$ is considered as a coalition, where $v({\mathcal {A}})$ explains the importance of coalition. Clearly, the strongest and weakest fuzzy measures are

7 $$\begin{aligned} v({\mathcal {A}})= \left\{ \begin{array}{ll} 1, &{} {\mathcal {A}}={\mathcal {N}}\\ 0, &{} \text {otherwise} \end{array} \right. \text {and} v({\mathcal {A}})=\left\{ \begin{array}{ll} 0, &{} {\mathcal {A}}=\emptyset \\ 1, &{} \text {otherwise} \end{array} \right. \text {, respectively.}\nonumber \\ \end{aligned}$$

The discrete Choquet integral with respect to a fuzzy measure *v* is given by Grabisch (2016); Grabisch et al (2009)

8 $$\begin{aligned} C_v(\pmb {x})= \sum _{i=1}^{n} x_{(i)} \Big [v \big (\{j | x_j \ge x_{(i)} \}) - v (\{j | x_j \ge x_{(i+1)} \} \big ) \Big ]\nonumber \\ \end{aligned}$$

where $\Big (x_{(1),}x_{(2),}\ldots x_{(n)}\Big )$ is a permutation of non-decreasing values and $x_{(n+1)}=\infty$. An alternative expression, more suitable for computing is

9 $$\begin{aligned} C_v(\pmb {x})=\sum _{i=1}^{n} \big [x_{(i)}-x_{(i-1)} \big ]v(H_i) \end{aligned}$$

where $x_{(0)}=0$ and $H_i=\{(i),\ldots ,(n)\}$ is the subset of indices of the $(n-i+1)$ largest components of vector $\pmb {x}$.

The variations in fuzzy measures allow the class of Choquet integral to include arithmetic mean, weighted arithmetic mean, OWA functions, as well as minimum, maximum, and order statistics as special cases.

Fuzzy measure is symmetric when

$$\begin{aligned} \text {if } |{\mathcal {A}}|=|{\mathcal {B}}| \text { then }v({\mathcal {A}})=v({\mathcal {B}}) \end{aligned}$$

holds.

Fuzzy measure is additive when

$$\begin{aligned} v({\mathcal {A}}\cup {\mathcal {B}})=v({\mathcal {A}})+v({\mathcal {B}}) \end{aligned}$$

holds.

When the measures are additive and symmetric, we get the arithmetic mean. When the measures are additive, but not symmetric, we get the weighted arithmetic mean.

When a coalition has a greater effect than the sum of its parts, the measure is super-additive:

$$\begin{aligned} v({\mathcal {A}}\cup {\mathcal {B}})\ge v({\mathcal {A}})+v({\mathcal {B}}). \end{aligned}$$

In the opposite case, the measure is sub-additive, i.e.,

$$\begin{aligned} v({\mathcal {A}}\cup {\mathcal {B}})\le v({\mathcal {A}})+v({\mathcal {B}}). \end{aligned}$$

The afore-explained properties of measures assume that the intersection of sets ${\mathcal {A}}$ and ${\mathcal {B}}$ is empty.

When one has a larger number of coalitions, it is possible to model that for several coalitions the whole is less than the sum of its parts, for the other subsets of coalitions the whole is more than the sum of its parts, whereas for the remaining ones the whole is equally relevant as the sum of its parts.

In addition, it is possible to model so-called total ignorance - there is no proper subset of groups with nonzero measures, whereas the coalition of all groups has a measure equal to 1 (Keller et al 2016). In the opposite case, all proper subsets of groups get a measure equal to 1, we model total confusion (see (7)).

Non-additivity allows flexibility in modeling the relevance of diverse coalitions. The only requirement is the monotonicity of fuzzy measure, i.e.,

$$\begin{aligned} \text {if }{\mathcal {A}} \supseteq {\mathcal {B}} \text {, then }v({\mathcal {A}})\ge v({\mathcal {B}}). \end{aligned}$$

Otherwise, the Choquet integral would not be an aggregation function. Let us recall that aggregation functions should meet three axioms: monotonicity, and two boundary conditions: $f(0,0,\ldots ,0)=0$ and $f(1,1,\ldots ,1)=1$. The boundary conditions are trivially satisfied, whereas for the monotonicity we need this property of fuzzy measure.

### Survey design: questions and answer options

With the whole universe of question types and having in mind capturing the hesitance, this work considers categorical or scaled questions aimed at expressing the degree of positive or negative opinion, or the degree of (dis)agreement related to a specific topic or statement. Thereby, the answer is a chosen value from the set of possible answers, for example, from intervals of integers [1, 5] or [1, 10], or categories expressed linguistically. On a five-level scale, it is conceivable to have categories like *clearly no*, *rather no*, *indifferent*, *rather yes*, *clearly yes* and the answer to a question can be one of these terms. The resolution of an answer can be more or less detailed. Generally, the number of categories should be within the range of three to nine, where nine is the upper bound for cognitive processing of information (Miller 1956). Yet, regardless the level of resolution, these scales do not cover respondents’ hesitation caused by various factors.

In this way, respondents usually choose answers from the predefined response scales, which speed up the answering process. More about the surveys can be found in, e.g., Hyman and Sierra (2016); Sue and Ritter (2012); Snijkers et al (2013); van Grinsven (2015); Wright and Marsden (2010). Open-ended questions require a deeper focus on constructing sentences, even though the hesitance can be freely expressed. On the other hand, it makes the computation costly. This paper does not concentrate on hesitance in open-ended questions.

To solve this problem, categorical questions with supporting questions about the respondent hesitance are merged. These questions are categorical as well. For instance, to get the most valuable information for city governance support, the respondents can express the hesitance in their answers caused by various factors (e.g., lack of information, experience, skills, feelings). For this reason, the answers are handled by a fuzzy set approach, i.e., a soft computing approach which is the most common mathematical tool for modeling uncertainty.

#### Example 1

Consider a simple questionnaire whose purpose is to gather information about the opinion of citizens living in a particular city district regarding the construction of a tram line in their area. Such a questionnaire could contain questions like:

*Would the new tram line lead to an increase in risk for other traffic participants, such as drivers or pedestrians?*

The respondent could then choose one of five possible answers indicating his or her agreement with the statement:

- 5 (*strongly agree*)
- 4 (*somewhat agree*)
- 3 (*I am indifferent*)
- 2 (*somewhat disagree*)
- 1 (*strongly disagree*).

The respondent is then also asked to express his or her level of hesitance, again by choosing one answer from a set of predefined answers:

*How confident are you with your answer?*

- *Very confident (0 - no hesitance)*
- *Somewhat confident (1 - some level of hesitance)*
- *My confidence is weak (2 - high level of hesitance).*

In the case of a clear non-hesitance, a crisp (precise) answer is expressed by a singleton fuzzy set (the support of fuzzy set between $a_{\tilde{A}}$ and $b_{\tilde{A}}$ collapses into a single value $m_{\tilde{A}}$, see Fig. 1 in Sect. 3.1. If the hesitance appears, then there is a TFN around the marked answer $m_{\tilde{A}}$. Thus, based on the hesitance level, it is more suitable to express the categorical answer by a fuzzy number.

In this way, the questionnaire design is tailored to the respondents groups (in terms of the number of questions and respective categorical answers) allowing them to express (real existing) opinions and feelings, which may motivate cooperation in surveys and mitigate non-responses rate (unit non-response and item non-response) and errors. In the next sections, the proposed aggregation model is introduced.

### Survey evaluation: aggregation process

After having defined collection opinions based on the above-mentioned questions and answer pairs, the next step is to choose an aggregation method. The aggregation operators reduce a set of values into a unique representation or meaningful number (Grabisch et al 2009). To aggregate answers from different stakeholder groups, the following three-level aggregation process has been proposed:

1. Aggregation of answers from a single respondent,
2. Aggregation of individual opinions into a single opinion of a specific group,
3. Aggregation of opinions considering relevance of various sets (coalitions) of stakeholder groups.

The results of the evaluation should get insight into the collected opinions and therefore support the decision-making at the city governance level. For this purpose, a three-level aggregation model (on the respondent level, on the group level, and among groups) is constructed.

#### Level 1: aggregation of respondent’s answers

At this level, the goal is to aggregate the answers provided by an individual respondent into a single fuzzy number, capturing both opinions and hesitance of all answers. Let assume the questionnaire contains questions like those presented in Example 1.

Each individual answer of a given respondent is first transformed into a triangular fuzzy number. Let $a_{ij}$ denote the answer of a respondent *i* on chosen discrete scale to answer *j* and $h_{ij}$ denote the level of hesitance on a scale from 0 to some $h_{\max }$ (in Example 1, it is value 2). The TFN $\tilde{a}_{ij}$ representing this answer can be then calculated as:

10 $$\begin{aligned} \tilde{a}_{ij}=\Big (\max \big (a_{\min }, a_{ij} - h_{ij}\big ); a_{ij}; \min \big (a_{\max }, a_{ij} + h_{ij}\big )\Big )\nonumber \\ \end{aligned}$$

where $a_{\tilde{A}}=\max (a_{\min }, a_{ij} - h_{ij})$, $m_{\tilde{A}}=a_{ij}$ and $b_{\tilde{A}}=\min (a_{\max }, a_{ij} + h_{ij})$, see Fig. 1.

It is clear from Eq. (10) that in the case of no hesitance, the fuzzy number collapses into a singleton (a single value $a_{ij}$). Moreover, the support interval is limited by the minimal and maximal values of the answer scale, denoted $a_{\min }$ and $a_{\max }$ respectively.

Individual fuzzy numbers are then aggregated using an appropriate aggregation function. Arithmetic mean is a suitable candidate as it is not biased toward a positive or negative opinion (Beliakov et al 2016). An example of a respondent-level aggregation based on arithmetic mean is shown in Table 1. Geometric mean, for instance, tends to bias the aggregation toward negative opinion, for scales where low positive numbers represent negative opinion and high positive numbers represent positive opinion. Contrary, the quadratic mean biases the aggregation toward positive opinion (Beliakov et al 2016).

When importance (or weight) is assigned to individual questions, the aggregation mechanism can be straightforwardly extended by the weighted arithmetic mean. Weights are either real numbers or fuzzy numbers. The decision depends on a task, whether it is more suitable to express the weights numerically or linguistically. Both cases can be easily added into the calculations. In the case of the latter, a multiplication of TFN expressing the weight and TFN explaining the answer is calculated by Eq. (2).

**Table 1:** Structure of answers and solutions for respondents in a group

Resp.	Question 1			$\dots$	Questions			Resulting TFN
	Answer	Hesitation	TFN		Answer	Hesitation	TFN	
R1	$a_{11}$	$h_{11}$	$\tilde{a}_{11}$	$\dots$	$a_{1s}$	$h_{1s}$	$\tilde{a}_{1s}$	$\tilde{A}_1$
R2	$a_{21}$	$h_{21}$	$\tilde{a}_{21}$	$\dots$	$a_{2s}$	$h_{2s}$	$\tilde{a}_{2s}$	$\tilde{A}_2$
R3	$a_{31}$	$h_{31}$	$\tilde{a}_{31}$	$\dots$	$a_{3s}$	$h_{3s}$	$\tilde{a}_{3s}$	$\tilde{A}_3$
$\dots$	$\dots$	$\dots$	$\dots$	$\ddots$	$\dots$	$\dots$	$\dots$	$\dots$
$\text {R}_{n}$	$a_{n1}$	$h_{11}$	$\tilde{a}_{n1}$	$\dots$	$a_{ns}$	$h_{ns}$	$\tilde{a}_{ns}$	$\tilde{A}_n$

This level is considered the preparation step for the next levels. In this step, the overall opinion about the investment plan, for instance, by each respondent is computed. The next step is devoted to the calculation of each stakeholder group opinion.

#### Level 2: aggregation within groups

The outcome of the first level is expressed by TFN covering the answers and hesitance in providing answers for each respondent. Having in mind that the respondents groups are imbalanced in the number of respondents, the number of questions, and when required, the different granularity of answers. Under these conditions, the overall opinion of each respondent group should be aggregated (Rakovská and Hudec 2019).

This level of aggregation calculates the relevance of a considered topic for a single respondent group by the relative quantifier *most of* of the structure *the most of respondents has positive opinion regarding the considered topic*. In this flexible aggregation way, the influence of careless answers is mitigated (i.e., respondents who provide neighboring values instead of desired ones are evaluated similarly as similar values have similar membership degrees to the fuzzy concept).

**Fig. 3:**
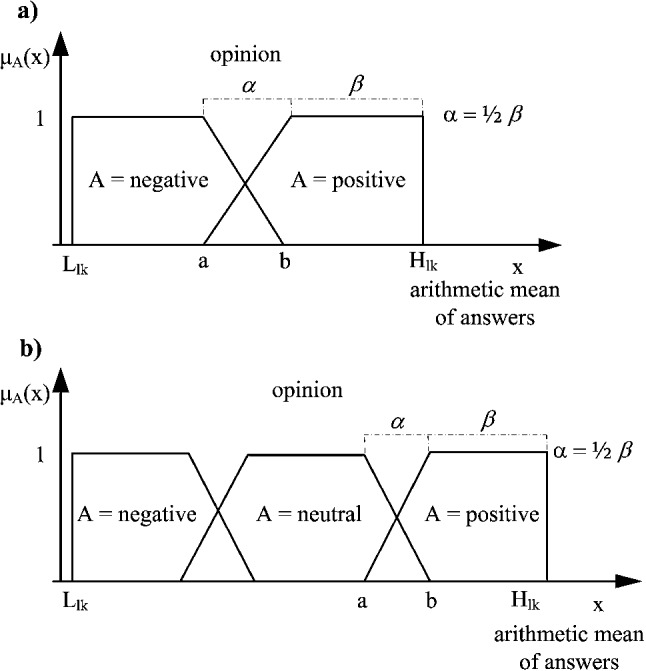
The linguistic variable *opinion* and its labels expressed by fuzzy sets, where $L_{lk}$ and $H_{lk}$ are the minimal and maximal possible values of the average of respondents’ answers expressed as singletons, respectively, a) linguistic variable consisting of two labels, b) linguistic variable consisting of three labels

The *positive opinion* is expressed as a fuzzy set, which is an element of a family of fuzzy sets covering linguistic variable *opinion* (see Zadeh (1975) for details about Linguistic Variables (LV)). In this context, LV consists of two labels: *negative* and *positive*, where an uncertainty area is around value 5. This value indicates the maximal uncertainty point between the clear negative (1) and positive opinion (10). The linguistic variable and its two labels are illustrated in Fig. 3a where in this case, $a = 4$ and $b = 6$. The maximal and clear negative opinion is singleton 1, whereas the clear positive opinion is singleton 10. By this interpretation, when value moves from *b* to *a*, the intensity of belonging to the *positive opinion* decreases. Thus, the smooth transition from the clear belonging to the clear non-belonging is managed. The *positive* and *negative* granules are constructed as linguistic labels of LV *opinion* using the *computing with words* concept (Zadeh 1996) (based on the fuzzy set theory) and applying the uniformly covering domain method (Tudorie 2008). For the categorical answers consisting of higher number of values (seven and more), it is better to construct LV *opinion* composed of three labels, i.e., *negative*, *neutral*, and *positive* as it is shown in Fig. 3b.

The solution from the previous step (see Sect. 3.5.1 and Eq. (10)) is a fuzzy number for each respondent. Thus, the possibility measure in Eq. (4) is applied to calculate the conformance between the aggregated respondent’s answers from Table 1 and the concept *positive opinion* by Eq. (10). For instance, Eq. (11) expresses the possibility degree that the aggregated answer of the *i*-th respondent is compatible with the fuzzy set *positive opinion* $\tilde{PO}$.

11 $$\begin{aligned} \text {Pos}(\tilde{A}_i,\tilde{PO}) = \sup _{x \in X} \min \big (\mu _{\tilde{A}}(x),\mu _{\tilde{PO}}(x)\big ) \end{aligned}$$

**Fig. 4:**
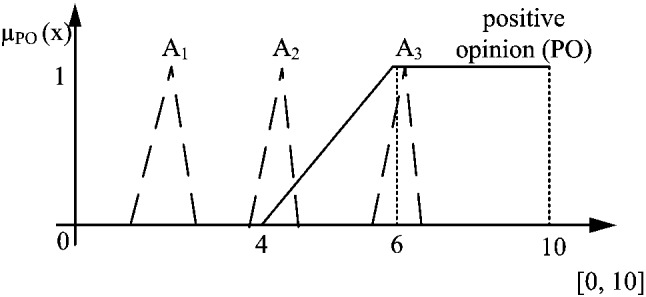
Possibility measure of three answers to the concept *positive opinion*, $P(\tilde{A}_1, \tilde{PO}) = 0, P(\tilde{A}_2,\tilde{PO})=\alpha$ and $P(\tilde{A}_3, \tilde{PO}) = 1$

The calculation is graphically illustrated in Fig 4. A convenient way of computation is through finding the maximal value of the intersection of a fuzzy number and fuzzy opinion as

12 $$\begin{aligned} \frac{b_{\tilde{A}} - x_{o}}{b_{\tilde{A}} - m_{\tilde{A}}}=\frac{x_{o}-a_{\tilde{PO}}}{b_{\tilde{PO}}-a_{\tilde{PO}}} \end{aligned}$$

when $m_{\tilde{A}}<b_{\tilde{PO}}\wedge b_{\tilde{A}}>a_{\tilde{PO}}$, where $a_{\tilde{PO}}=a$ and $b_{\tilde{PO}}=b$ in Fig. 3. The point where the intersection appears, is

13 $$\begin{aligned} x_o=\frac{a_{\tilde{PO}}m_{\tilde{A}}-b_{\tilde{A}}b_{\tilde{PO}}}{(a_{\tilde{PO}} -b_{\tilde{PO}})+(m_{\tilde{A}}-b_{\tilde{A}})} \end{aligned}$$

which correlates with the calculations examined by Galindo et al (2006). The matching degree of possibility in Eq. (11) is calculated by assigning $x_o$ into the membership function for fuzzy number or fuzzy concept.

Clearly, when $m_A\ge b_B$ the solution is 1 and when $b_A\le a_B$ the solution is 0. Formally, the calculation is expressed as

14 $$\begin{aligned} \text {Pos}(\tilde{A},\tilde{PO})=\left\{ \begin{array}{ll} 1, &{} m_{\tilde{A}}\ge m_{\tilde{PO}}\\ \mu _{\tilde{PO}}(x_0), &{} m_{\tilde{A}}<b_{\tilde{PO}}\wedge b_{\tilde{A}}>a_{\tilde{PO}}\\ 0, &{} b_{\tilde{A}}\le a_{\tilde{PO}}\\ \end{array}\right. \end{aligned}$$

where $x_0$ is calculated by Eq. (13). An illustrative example for several fuzzy numbers is shown in Fig. 4.

Consequently, by Eq. (5) and (6), the validity of the sentence *most of the respondents have a positive opinion regarding the considered topic* for each respondent group is calculated. The result of this aggregation is in the unit interval, indicating the intensity of positive opinion of the whole group. Therefore, in the next aggregation level, any aggregation function can be assigned without scaling to the unit interval.

**Fig. 5:**
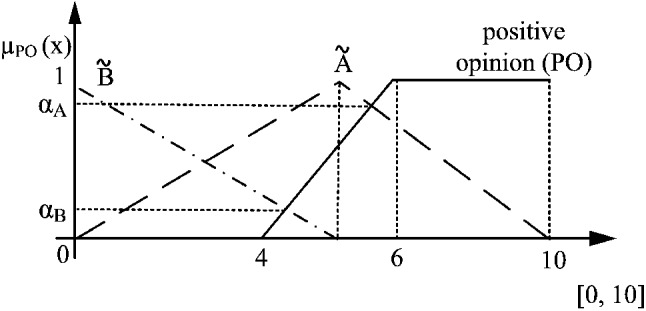
Possibility measure of the neutral answer with maximal hesitance

The occurrence of the highest level of hesitance should be limited as managed by Eq. 10. Otherwise, if the answer is *do not know* with the highest hesitance, a high value of the possibility measure to the concept *positive opinion* is calculated as demonstrated in Fig. 5.

A TFN must therefore have a limited support. Imagine a fuzzy number *altitude above sea level around 3 000 m* with the highest hesitance of the limiting values $a_{\tilde{A}}$ and $b_{\tilde{A}}$. In this case, the support ($\text {Supp}(FM)={x|x\in {\mathbb {X}}\wedge \mu _{FM}(x)>0}$) covers the whole domain (from the Dead Sea to Himalayas). Clearly, it is not a suitably constructed fuzzy number, even though the user hesitance is high. The same observation is adopted for a fuzzy number covering the maximal hesitance in user opinions. This problem has been solved by the limited support of hesitance applying Eq. (10). The answers with no hesitance are considered as classical numbers or singletons.

The next section is focused on the calculation of the aggregated opinion considering all stakeholder groups by the aggregation based on the Choquet integral.

#### Level 3: aggregation among stakeholder groups

A short reminder of the aggregation function categorization. The four main classes of aggregation functions are due (Dubois and Prade 2004):

- Conjunctive characterized by $0\le A(\pmb {x})\le \min {(\pmb {x})}$
- Disjunctive characterized by $\max \le A(\pmb {x}) \le 1$
- Averaging characterized by $\min (\pmb {x})\le A(\pmb {x})\le \max {(\pmb {x})}$
- Remaining aggregation functions are called mixed

where $\pmb {x}$ is a vector $\pmb {x} = (x_1, \ldots , x_n)$.

When a considered topic should be positively evaluated by the majority of groups, disjunctive functions are out of question, because when only one group fully agrees and the other groups fully disagree, the solution is full satisfaction. Conjunctive functions might be applied (i.e., an investment plan to some extent positively evaluated by all groups), but the main problem is ignoring the higher values than the minimal one by the min t-norm (i.e., non-compensatory effect), or downward reinforcement by the other t-norms (Klement et al 2000), i.e., the aggregated value is lower than the minimal value given by all respondent groups. In practice, this may be interpreted as the topic has a low acceptable value among groups. Thus, the averaging functions seem to be best suited for the task at hand.

However, evaluating groups independently can be considered too simplifying. For instance, aggregation by arithmetic mean, when one group considers a project as fully unacceptable, while some other group considers the same project as perfectly acceptable, would result in a solution equal to the case when both groups consider the project acceptable with a medium degree. The other averaging functions like geometric or harmonic means also consider all groups independently. The weighted cases distinguish the importance of stakeholder groups, but groups are still handled independently. It is, therefore, reasonable to consider other averaging functions. As already discussed, in the evaluation, some sets (or “coalitions”) of stakeholder groups are more relevant than the others.

Hence, for the third level the suitable aggregation is by the discrete Choquet integral (9).

##### Example 2

Consider four stakeholder groups in a city whose opinion about a specific project is being evaluated:

- L-citizens living in an affected district
- C-citizens commuting to this district
- T-professionals in transportation
- E-environmental activists

In total, there are 16 possible coalitions (set of groups) that can be created from these groups (including an empty coalition and a coalition of all groups). For example, the following degrees of importance were assigned to these coalitions: $v({\mathcal {N}})=1$

$$v(LCT) = 0.75, v(LCE) = 0.75, v(LTE) = 0.85, v(CTE) = 0.6$$

$$v(LC) = 0.65, v(LT) = 0.6, v(LE) = 0.65, v(CT) = 0.4, v(CE) = 0.4, v(TE) = 0.5$$

$$v(L) = 0.4, v(C) = 0.2, v(T) = 0.3, v(E) = 0.3$$

$$v(\emptyset )=0$$

Observe that the sets citizens living in the affected area (L) and citizens commuting (C) to the affected area are two sets with an empty intersection. However, together they create the most affected group, so the capacity is greater than the sum of their respective capacities. In this case, we have super-additivity. The opposite (sub-additivity) holds for merging groups of citizens commuting to the affected area (C) and ecological activists (E).

Note that when all stakeholder groups are equally important, $v(L) = v(C) = v(T) = v(E)$ we can apply the arithmetic mean. The evaluator, for instance, considers the coalition of citizens and environmental activists (when both agree) as more important than the coalition of citizens and transportation professionals, as well as the coalition of environmental activists and transportation professionals. As a rule, the importance of a coalition cannot be lower than the importance of any of its sub-coalitions. This clearly holds here, as, for instance, all single-member coalitions have a lower or equal importance than any two-member coalition, and the coalition of groups C, T, and E has a higher importance than the coalitions {C, T}, {C, E}, and {T, E}, but a lower importance than the coalition consisting of groups L, T and E.

Let’s compare the aggregation results in the two cases defined in Table 2. These cases differ in the permutation of the positively expressed degree toward the project by each group. The value of the discrete Choquet integral for both cases is calculated by Eq. (9), obtaining the solution 0.406 for case A and 0.482 for case B. Hence, the project would be considered more acceptable if the degree of positive opinion matched case B. This is not surprising, as much higher importance is given to coalitions containing group L and the degree of positivity in this group is much higher in case B. By applying arithmetic, geometric or quadratic mean, no difference is recorded, especially when each separate group has the same relevance, but their coalitions do not.

**Table 2:** An illustrative example of aggregation by the discrete Choquet integral in Eq. (9) for two cases of permuted groups’ matching degrees

Group	Case A	Case B
L	0.30	0.65
C	0.65	0.30
T	0.42	0.36
E	0.36	0.42

The next section illustrates the developed model on a case study in a city.

## Case study: building new tram Lines in city district

In this section, the authors provide a real-world numerical example demonstrating the applicability of the proposed evaluation model.

### Problem description

Ostrava is a city in the Czech Republic with approximately 300,000 inhabitants. Local representatives together with the local public transport company have come up with the idea of extending the tram line in the Poruba district, one of the largest districts in the city with 70,000 inhabitants. They present this idea as a significant innovation step in the field of mobility that will lead to a higher level of comfort for people and prosperity for businesses. However, very soon, this proposal gave rise to a great wave of opinions on various pros and cons from diverse subjects. Apparently, the view of innovation is not so united as the representatives expected. There are several groups of stakeholders following different goals. And these stakeholders provide different perspectives in their opinions. Namely, based on previous examinations done by the local government, the following groups of stakeholders have been identified:

- *People living in Poruba (IL)* It can be assumed that these people will be primary users of the new tram line. On the other hand, they can be also most negatively impacted by the innovation.
- *People living in other districts of Ostrava commuting to Poruba (IF)* This group is supposed to assess the innovation from the travelling comfort point of view, when going to work in Poruba or visiting families, friends, shops, and other places.
- *Environmentalists (EN)* The new track will inevitably impact the environment in the Poruba district. This impact must be evaluated by experts in this field to avoid irreversible damage to the ecosystem.
- *Managers of the local shops and service providers (MA)* The business sector is also an essential stakeholder of the district’s development. Urban development can be vital for further investment and growth of companies.
- *Urban architects (UA)* Architects should be able to assess the planned project more objectively and from a longer-term perspective.

It is natural that the set of questions is not the same for all groups, because they are driven by different utility functions, values, experiences, needs, and concerns, see Table 3. All questions can be answered using the five-point scale where degree 1 belongs to the answer absolutely supporting the track innovation. The other way around, degree 5 corresponds to the answer, which is absolutely against the track. It is worth mentioning that the answer “yes” can mean both approval and disapproval, depending on the given question. For the sake of clarity, the meaning of degree 5 is explained for each question in the last column of Table 3. The middle degree (3) is used to express the *I do not know* opinion.

This way of designing questions does not affect the calculations introduced in Sect. 3, because the definitions of *positive* and *negative* opinion concepts can simply be switched (see Fig. 3 in Sect. 3.5.2). The questions were chosen based on the topics which were most frequently discussed during the local government meetings so far.

**Table 3:** The list of questions for the included stakeholders

Question	Group	Degree 5
Will the new track decrease the safety of traffic (including the pedestrians)?	IL	Absolutely yes.
Will the new track decrease the comfort of living for people living close to the track?	IL, IF, UA	Absolutely yes.
Will the new track decrease the your comfort of living?	IL	Absolutely yes.
Do you consider the current state of the public transport in Poruba satisfying?	IL, IF, UA	Absolutely yes.
Will you be more motivated to come to Poruba more often for your leisure time activities?	IF	Absolutely no.
Will you be more motivated to come to Poruba more often for shopping and services?	IF	Absolutely no.
Will the new track attract you to move to Poruba?	IF	Absolutely no.
Will the new track play the vital role for further development of the district?	UA	Absolutely no.
Will the new track negatively influence the landscape and urban character of the district?	UA	Absolutely yes.
Do you consider the current state of the public transport in Poruba satisfying for the next 10 years?	UA	Absolutely yes.
Will the new track damage the local biotope?	EN	Absolutely yes.
Will the new track bring the substantial loss of public greenery?	EN	Absolutely yes.
Will the new track be a real threat for local endemic species?	EN	Absolutely yes.
Will the new track motivate you to further investment in your business?	MA	Absolutely no.
Will the new track increase the attractivity of your business in Poruba?	MA	Absolutely no.

In line with the methodology described in Sect. 3, all stakeholders were also asked to describe their hesitance level (0 = no hesitance; 1 = weak hesitance, more data and information are required to be sure; 2 = strong hesitance, opinion driven rather by intuition).

The questionnaire was distributed among the subjects partly by e-mail, partly in a face-to face way. It is worth noting that the selection of the interviewees was not done completely randomly, but it was strongly influenced by available contacts. Thus, the results can be biased. On the other hand, the goal of this case study is to demonstrate the applicability of the proposed methodology, and the results themselves are in this context secondary. Nevertheless, the evaluations were made as unbiased as possible.

The final dataset^1^ contained 31 responses from the IL group, 22 responses from the IF group, 6 responses from the UA group, 9 responses from the EN group, and 12 responses from the MA group.

### Data processing and results

The proposed three-level aggregation model was implemented in two Jupyter notebooks^2^ using Python and its commonly used scientific libraries (NumPy and Matplotlib).

**Fig. 6:**
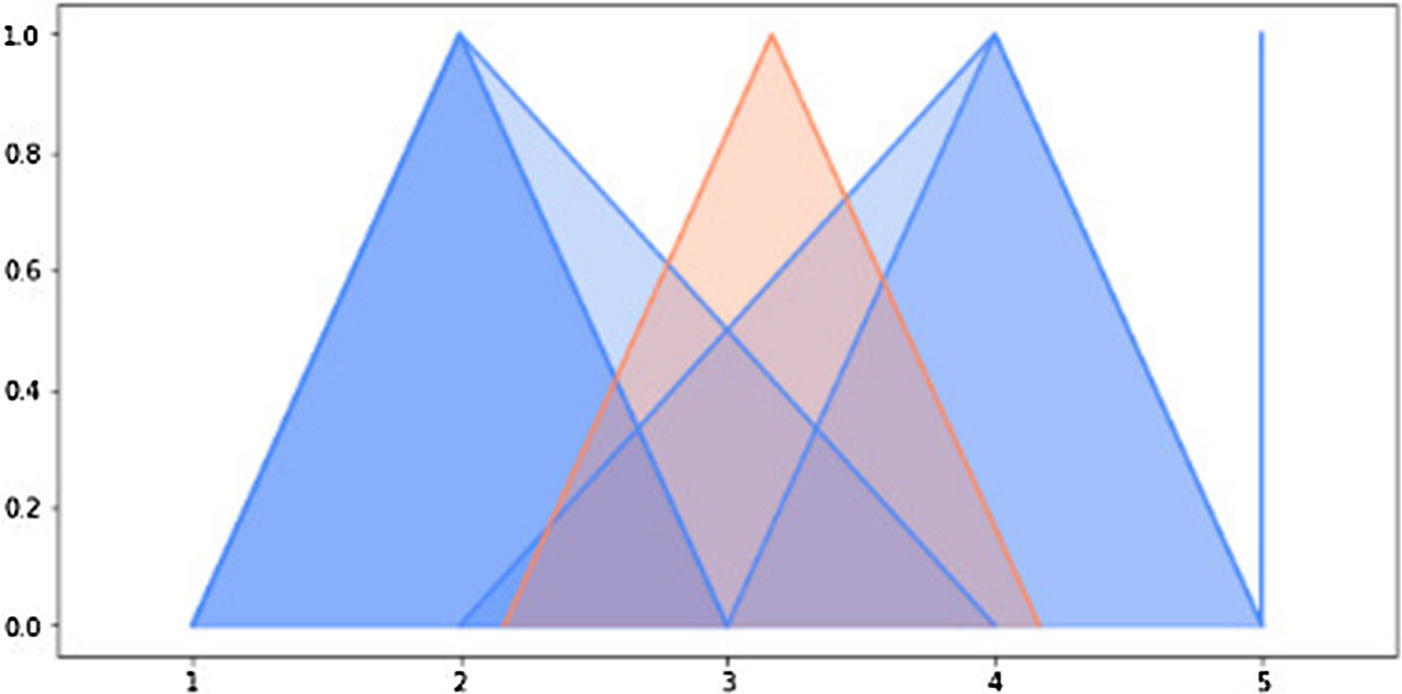
Aggregation of answers (blue) from a single respondent using arithmetic mean (orange) (color figure online)

For the first level of aggregation, the implementation is straightforward. Using Eq. (10), the answers are first transformed into a triangular fuzzy number (or a singleton) and then the arithmetic mean of fuzzy numbers is calculated with Eqs. (1) and (3). The results are visualized for each respondent. For instance, the answers of a single respondent from the IF group and their aggregation are shown in Fig. 6.

**Fig. 7:**
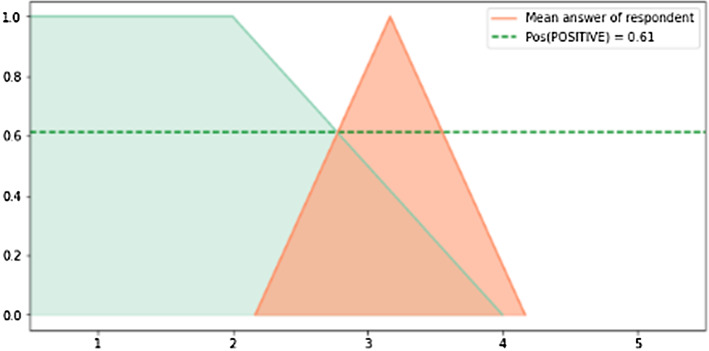
Degree of conformity with a fuzzy set expressing positive opinion for a single respondent

At the second level of aggregation, the process is started by calculating the degree of conformity of each respondent with the fuzzy set representing *positive opinion*. In the case study, it is a trapezoidal fuzzy set, with support [1,4] and core [1,2]. Given this definition, the level of conformity is then calculated using Eq. (14). The visualization of the conformity for the same respondent as in Fig. 6, is shown in Fig. 7.

After the degrees of conformity are established for all respondents in a group, the proportion of positive opinions within this group is determined. It is calculated as a sum of all degrees of conformity divided by the number of respondents in the group. Finally, the validity of the aggregated summarized sentence *most respondents have positive opinion* is determined by Eqs. (5) and (6).

**Fig. 8:**
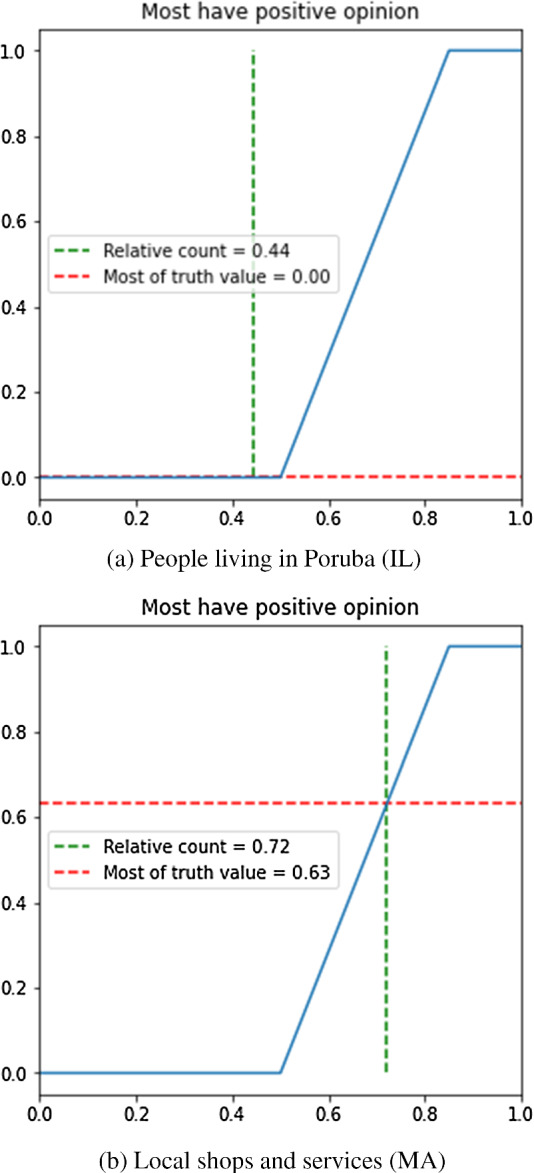
Results of the second level of aggregation

A graphical representation of the calculation for the IL and MA groups is shown in Fig. 8. Managers of local shops and services are relatively positive about the tram line project. They might expect the tram line to make their business more accessible for customers and make them a more attractive employer as commuting would become much easier. On the other hand, the citizens of the Poruba district are not happy at all. The reasons might include the perceived negative impact on traffic safety, level of noise, or distortion of the district’s relatively “green” landscape.

Complete results of the second level of aggregation for each stakeholder group, are provided in Table 4. For the sake of comparison, the degree of negative opinion is also calculated for each group, with one difference - the fuzzy set with support [2, 5] and core [4, 5] (Fig. 3) is used instead. Most groups undoubtedly have a negative opinion on the project except for local companies. Then, the opinion of environmental activists seems to be very negative, while the level of negativity is only mild among urban architects. For the IL and MA groups analyzed above, the degree of negative opinion provides additional support for the views on the impact of the new tram line described above.

**Table 4:** Second level of aggregation results for each stakeholder group

Group	Degree of positive opinion	Degree of negative opinion
IL	0	0.60
IF	0	0.68
EN	0	0.84
MA	0.63	0
UA	0	0.30

These results are used as input for the second Jupyter notebook, whose task is to calculate the discrete Choquet integral, hence determine the overall degree of positive/negative opinion toward the new tram line.

To calculate the discrete Choquet integral, the importance of different coalitions among groups must be established. For the sake of illustrating the applicability of the three-level aggregation model, subjective opinions and experience of the authors were applied, favoring coalitions including the group of Poruba citizens (IL). The calculation itself is based on Eq. (9).

Given the results of the second level of aggregation, the resulting values are 0.19 and 0.65 as the consensus on the level of positivity and negativity, respectively. Hence, the conclusion is that the overall perception of the new tram line is negative. In this context, the city governance has to decide if to implement the proposed innovation even if the majority has negative opinions about it, or to consider a modified route.

Next, the Choquet integral output is compared with the results produced by the simple arithmetic mean. Two scenarios are considered: (a) answer hesitance taken into account and (b) answer hesitance ignored. All situations are captured in Table 5.

**Table 5:** Comparison of aggregation results of the Choquet integral with arithmetic mean with and without hesitation taken into account

	With hesitance		Without hesitance	
Group	Pos.	Neg.	Pos.	Neg.
IL	0	0.6	0	0.35
IF	0	0.68	0	0.45
EN	0	0.84	0	0.91
MA	0.63	0	0.56	0
UA	0	0.3	0	0.16
Choquet	0.19	0.65	0.17	0.52
Arithmetic mean	0.126	0.484	0.112	0.374

When aggregating with Choquet integral, the overall degree of negative opinion was higher compared to the arithmetic mean. This result is consistent with the fact that people living in Poruba have a high degree of negative opinion and coalitions that include this group are considered as more important. A similar effect can be seen for the degree of positive opinion.

Similarly, in scenarios with hesitance taken into account, the overall degree of positive and negative opinion was much greater. A possible explanation might be that incorporating hesitance allowed the model to capture small bits of positive (negative) perception even when the overall opinion of a respondent was relatively negative (positive).

These results could have been significantly different if the coalition weights, expressing their importance, were set differently. An investigation of the robustness of the proposed three-level aggregation model is an open question for future research, see the discussion in Sect. 5.

## Discussion

This section discusses the proposed model and its applicability in various fields. One of the core tasks is, among others, to identify topics which touch diverse stakeholder groups, topics that are considered mutually beneficial, or to get feedback regarding investment plans like new tram lines or extensive housing construction.

Thanks to its flexibility, the model can be easily applied in tasks where only the first and the second level of aggregation are sufficient. The domain of medicine is a possible example. Patients cannot always precisely explain the feelings or the intensity of pain. The first level can be adopted to questions related to the medical examination of each patient. If the second level is included, the structured answers can be further evaluated to obtain either a general overview or an overview among different categories of patients (e.g., by gender, occupation, and the like).

The case study demonstrated how diverse is the perception of the new tram line proposal among different groups of stakeholders. The identified stakeholder groups are not seen as exclusive. It is always possible, in a further iteration, to add, remove or merge stakeholder groups if the context changes.The second level reveals group(s), where most of the members feel discomfort. In this case study, it holds for the citizens living in the affected area. Thus, local authorities should take the chance to find a dialog with the stakeholder groups, especially with those who are against the innovation. Next, the third aggregation level reveals the overall conformance with the plan. It gets values from the unit interval, i.e, ranked from 0 (not at all) to 1 (perfect).

Moreover, when a city wishes to evaluate public transport line proposals in various districts, each district can be evaluated independently by its own stakeholder groups. Afterwards, they are ranked downwards from the district with the highest consent by the result of the third aggregation level. In contrast, pairwise comparison might be tricky in such case. Although the level of consent within the winner might be the highest among its “competitors”, it can be still relatively low in absolute terms. The low value on the third level (considering the relevance of each group and coalitions among groups) is a signal to the city governance that a new transportation plan should be improved or better communicated across the entire set of urban stakeholder groups.

From a more technical point of view, the model addresses the issues of unequal sizes of groups (reflected in different number of filled questionnaires) and tailored questions, uncertainty and hesitance in answers, and various levels of relevance of so-called coalitions of groups. The problem of uncertainty and hesitance from respondents is addressed by modeling each answer as a TFN. In the case of no hesitance, the usual scaled question from the Likert scale (or a variant thereof) can be used. The coalitions among groups are covered by the discrete Choquet integral. When all groups are considered as fully independent, any averaging function can be applied. However, a care should be taken when the arithmetic mean is applied due to its full-compensation effect. When all groups should at least partially agree, then the geometric mean is a solution (an averaging function with 0 as an annihilator). Theoretically, the other averaging functions meeting the property of annihilator could be applied.

This work proposes an enhanced evaluation model to support decision-making considering uncertainty and hesitance, a natural human feature in expressing their opinions.

Furthermore, the proposed three-level aggregation model can also be used in other fields, where hesitation in opinions and peculiarities of diverse respondent groups should not be neglected. An illustrative example is the online teaching evaluation during the pandemic situation. The Ministry of Education or other relevant institutions can be interested in the opinions among universities covering groups like teachers, students, or technical IT staff. In the case of evaluation at elementary schools, parents, pupils, teachers, or technical IT staff are the groups of interest. Considering pupils, a higher level of hesitance is expected as they usually are not able to clearly express their opinions. Questionnaires for pupils should be very carefully designed. It is a topic for experts in didactics and related fields. However, when the questionnaire is built, opinions can be straightforwardly included into the model. Next, parents and pupils can be naturally considered one of the coalitions.

Moreover, the first aggregation level can be used independently when opinions (considering hesitance) are collected from a single group of respondents. In this case, well-established statistical functions and logic aggregation functions can be applied to fuzzy numbers. The opinion of a respondent expressed as TFN (last column in Table 1, Sect. 3.5.1) can be defuzzified into a real number by one of well-established defuzzification methods for TFN (see, e.g., Hudec (2016)), and further evaluated.

A disadvantage of the artificial degrees like the one used in the presented model is the fact that it is qualitative, as well as the original evaluations of respondents. On the one hand, it is reasonable to make a qualitative conclusion from the original qualitative evaluations. On the other hand, the interpretation of the result and the final recommendation can sometimes be uneasy. It depends on the problem which is surveyed. For instance, if a university explores the overall satisfaction of students with teaching, the resulting degrees of negative and positive opinions (i.e., satisfaction) provide a satisfactory summarizing perspective on the issue accompanied with some feelings. Different situation occurs when the survey is performed to decide about performing some action. For example, a local government has to decide how to utilize an old production hall in the city centre and two options (A and B) are under consideration. One of them must be chosen. For the sake of simplicity, let us assume that the degree of positive opinion for A equal to 1 and negative opinion for A equal to 0 mean that people absolutely agree with A and absolutely disagree with B at the same time (and vice versa). However, it is hard to even imagine that the results would be so unambiguous. It can happen that both degrees can be quite similar or even the same (e.g., equal to 0.5). In such case, it is very difficult to make a final recommendation. The stochastic evaluation would provide statistical tests, revealing if the difference between the values is statistically significant. Unfortunately, no similar tool for significance testing is available here. Therefore, it cannot be recommended to take a decision without any further supporting analysis. First, the robustness of the results can be checked by varying the weights of the coalitions used for the Choquet integral. Let us recall that these weights are subjective and expertly set, thus it is reasonable to explore how much stable the solution is for slight changes in these weights. Second, another round of the survey can be run with some additional questions or some additional information can be provided to the respondents to (a) decrease their hesitance, (b) make their evaluations more distant from the neutral opinion.

It is worth noting that the impact of the degrees of positive and negative opinions on the final recommendation can also be asymmetric. For example, the degree of positive opinion for building a new tram track in the city is equal to 0.6, meanwhile the degree of negative opinion is equal to 0.4. Despite the positive opinion is by 50% stronger, it does not necessarily result in the recommendation that the track should be built. The reason is analogical to the focal point (also called Shelling point) in game theory, see Webster (2014). When one is deciding whether to keep the current state or make a change, the motivation for the change must be substantially stronger than for staying in the current position (because of convenience, fear of uncertainty, and other factors). In this case, some threshold values for the resulting degrees can be adopted, but it is questionable how to set them.

To conclude, the proposed model provides just the support for the future decision. However, this support should be considered a hint which should be assessed expertly before providing the final recommendation. Despite no universal manual what to do about the resulting degrees of satisfaction can be provided, the framing overview revealed by the proposed model can be beneficial.

## Conclusion

When collecting opinions from diverse respondent groups, we face the problems of unbalanced size of groups, different background of groups, and the relevance of the subsets of various groups. To contribute, we raised the research questions of handling hesitance in answers, aggregating answers within non-balanced groups, and aggregating among groups (considering the relevance of subsets of groups). The question is, whether they can be solved by fuzzy logic and aggregation functions. The answer is that it is possible using the three-level model of opinion aggregation which manages hesitance and unbalanced groups of respondents. In this direction, we extended the previously proposed model developed by Rakovská and Hudec (2019) by considering hesitance and implementing the third level of aggregation using the discrete Choquet integral. This extended model was then applied to a case study on evaluating a new tram line.

In the literature, studies are mainly focused on opinion collection, either within smaller expert groups, or on surveys considering the whole population (or a representative sample), without dividing respondents into groups, considering their background and how specific subgroups are affected by the topic of survey. We have softened the gap between these two edge cases by the proposed three-level model for opinion aggregation under hesitance.

In the case study evaluating a new tram line, the collection and evaluation of surveyed data covered these aspects, namely:

- Different level of skills and views among respondent groups (e.g., citizens living in a district, citizens commuting to a district, transportation experts, ecologic activists),
- Hesitance in answers,
- Different relevance of coalitions and subsets of groups (i.e., high agreement in answers of citizens living in an examined area and ecological activists is not the same as consensus between commuters to the considered area and transportation experts).

At the first level (respondent level), the arithmetic mean of triangular fuzzy numbers (expressing vagueness and hesitance in a categorical answer) is applied to compute the respondent’s overall opinion. At the second level, the possibility measure calculates the conformance of the overall respondent’s answers with the concept *positive opinion*, whereas quantified aggregation by the fuzzy quantifier *most of* calculates the conformance of the respondents group with the concept *positive opinion*. Finally, at the third level, the importance of coalitions is managed by the discrete Choquet integral and fuzzy measures.

Constructing a new tram line, or other projects in cities, are financially demanding. A higher level of consensus among the stakeholders groups is an additional support for this investment. In the opposite case, local government should consider alternatives or open further discussions with citizens and other affected groups.

The proposed model can be applied in diverse fields. For instance, one can evaluate distant learning at universities or elementary schools. In education, categories of respondents like students (or pupils and parents), teachers, and IT staff can be recognized. Another current issue is the motivation for immunization and observance of measures ordered by epidemiologists and the government. A survey might reveal which category of respondents is strongly against immunization (or find measures against spreading of virus which are irritating and annoying). Governments will be able to recognize these categories and adjust the vaccination strategy or prepare the targeted campaign. For complex applications, a cooperative effort of experts and researchers working in the domain of interest, soft computing, respondent motivation, adaptive survey design, and other related fields is welcome. With the increasing acceptance and maturity of computational intelligence, new possibilities are arising in the area of human-machine interaction.

It is worth emphasizing that the proposed model should not be considered as a competitor to the existing ones, but rather as a complementary survey approach to well-established ones for a class of opinion collection and analyzing problems.

## Data Availability

The datasets generated during and/or analyzed during the current study are available in the github repository, https://github.com/syseng-ostrava/3-level-opinion-aggregation/tree/master/data. Then, the used Python codes are also available in the github repository, https://github.com/syseng-ostrava/3-level-opinion-aggregation/tree/master/notebooks.

## Footnotes

1 https://github.com/syseng-ostrava/3-level-opinion-aggregation/tree/master/data.

2 https://github.com/syseng-ostrava/3-level-opinion-aggregation/tree/master/notebooks
